# Synthesis and RP HPLC studies of biologically active semicarbazides and their cyclic analogues 1,2,4-triazol-3-ones

**DOI:** 10.1007/s00706-011-0715-z

**Published:** 2012-01-28

**Authors:** Monika Pitucha, Joanna Matysiak, Bogdan Senczyna

**Affiliations:** 1 Department of Organic Chemistry, Medical University of Lublin, Lublin, Poland; 2 Department of Chemistry, University of Life Sciences in Lublin, Lublin, Poland

**Keywords:** Semicarbazide, 1,2,4-Triazol-3-one, RP HPLC, IAM chromatography, Lipophilicity

## Abstract

**Abstract:**

The retention behaviour of semicarbazides and their cyclic analogues 1,2,4-triazol-3-ones, has been investigated by RP-8, RP-18 and IAM HPLC. The structures of new derivatives were proved by elemental analyses, IR, ^1^H NMR and ^13^C NMR spectroscopy. The compounds showed regular retention behaviour in three chromatographic systems; their log *k* values decreased linearly with the increasing concentration of an organic modifier in the mobile phase. The ratio of the intercept (log *k*_w_) to the slope of compounds is constant and the same for both groups of compounds on C18 and IAM stationary phases. Differences between log *k*_w_ values from the octadecyl stationary phase of corresponding cyclic and linear derivatives are constant, and they are related to the mechanism of synthesis of 1,2,4-triazol-3-ones from linear substrate semicarbazides, which was confirmed by modelling studies. Good correlations between log *k*_w_ parameters obtained by RP-8 or RP-18 and determined by the computational approach log *P* were found.

**Graphical Abstract:**

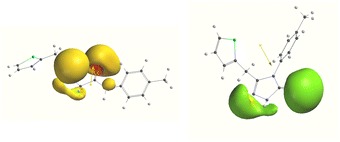

## Introduction

Triazoles and their heterocyclic derivatives represent an interesting class of compounds possessing a wide spectrum of biological activities [1–4], including antibacterial ones [5, 6]. Examples of drugs bearing the 1,2,4-triazole residue are the powerful azole antifungal agent fluconazole [7], as well as the potent antiviral *N*-nucleoside ribavirin [8].

Their linear analogues, semicarbazides, commonly applied in the 1,2,4-triazole synthesis, are also an important class of compounds of diverse biological properties. They have been studied as anticonvulsant [9], antitubercular [10] and antinociceptive [11] agents. Additionally, the derivatives having the heterocyclic ring exhibit antibacterial activity, inhibiting growth of some gram-positive bacteria, including *B. cereus* and *M. luteus* [12]. Some of them show antinociceptive activity in a wide range of doses [13].

It is commonly known that biological activity of compounds is a function of their lipophilicity. Reversed phase (RP) chromatographic methods have been extensively applied to determine this property of many bioactive compounds [14, 15]. The use of alkyl-bonded phases in liquid chromatography offers a convenient and highly accurate method for the evaluation of the lipophilicity of a large variety of compounds [16, 17]. As the HPLC stationary phase for the determination of phase affinity of compounds, the immobilized artificial membrane (IAM) is also used [18–20].

A linear relationship between the retention parameters (log *k*) and the concentration (*φ*) of organic modifier (acetonitrile, methanol, or others) in the aqueous mobile phase described by the Soczewiński-Wachtmeister equation (Eq. 1) has to be established for a successful chromatographic measurement of lipophilicity [21, 22].

1 $$ \log k = \log k_{\rm{w}} + S  \varphi $$

log *k*_w_ represents the retention factor of a solute with pure water as the mobile phase; *S* is the slope of the regression curve. This dependence allows for the extrapolation from the data obtained for the water-organic modifier to water as the mobile phase. On the basis of log *k* = f(*φ*) relationships, the lipophilicity parameter log *k*_w_ and the specific hydrophobic surface area *S* can be calculated. Lipophilicity chromatographic descriptors obtained in this way help explain differences in the bioactivity of similar structure derivatives. They are usually used for QSAR model building, prediction of biological activity of new structure related analogues, and design and synthesis of more effective new derivatives [23, 24].

In this work comparable chromatographic studies of biologically active mono- and bis-1,2,4-triazol-3-ones as well as their linear analogues, semicarbazides, were carried out. The octyl, octadecyl and IAM stationary phases were used. To explain differences in the retention behaviour of compounds of both groups, molecular modeling was performed. Log *P* values obtained from the computational approach were used comparatively.

## Results and discussion

**Scheme 1:**
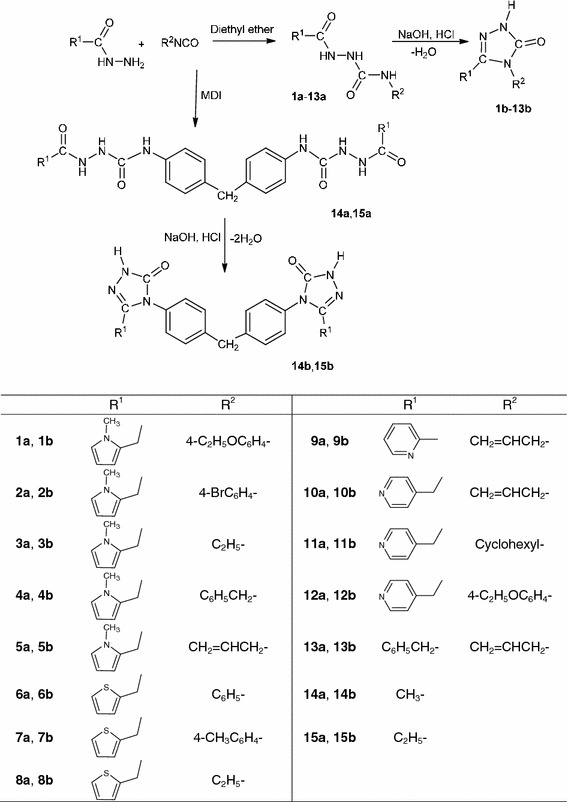

The structures of compounds under consideration are presented in Scheme 1. They include linear semicarbazides (**a**) and their cyclic analogues 3*H*-1,2,4-triazol-3-ones (**b**). The compounds of structure **b** were obtained by condensation reaction of **a** in alkaline medium. The process of water molecule elimination from compounds **1a**–**13a** or two molecules from **14a** and **15a** gives the corresponding 3*H*-1,2,4-triazol-3-ones **1b**–**15b.**

The substitution panel of compounds under consideration includes heterocyclic, aryl, alkyl and alkenyl groups. The structures of new derivatives were proved by elemental analyses, IR, ^1^H NMR and ^13^C NMR spectroscopy.

**Fig. 1:**
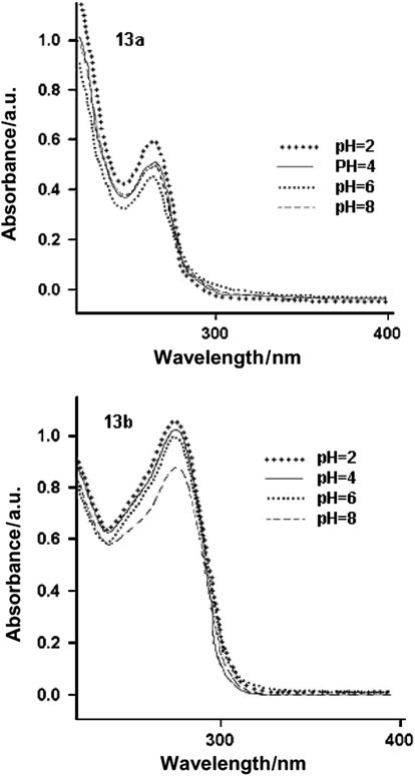
UV–Vis spectra of compounds **13a** and **13b** in methanol-water solution of different pH

The UV-Vis spectra in water-methanol solutions of different pH of compounds **13a** and **13b** are presented in Fig. 1. They show that there are no significant changes in their electronic structure at pH 2–8. As pH 7.4 (physiological) is recommended for IAM chromatography measurements, for comparison all chromatographic experiments were performed at this pH.

**Fig. 2:**
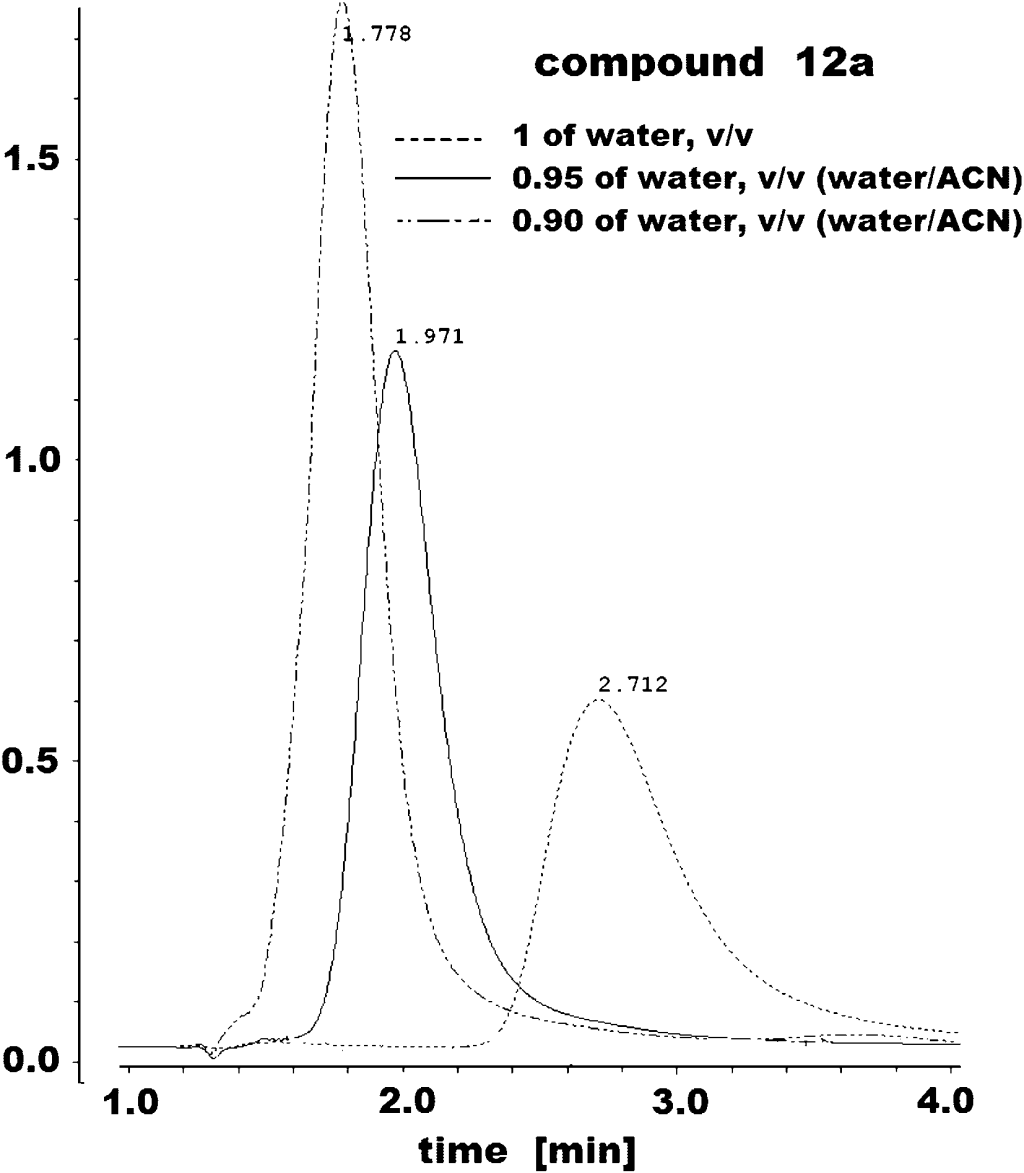
Chromatograms of compound **12a** obtained by IAM chromatography for different water contents in water/ACN mobile phase

The log *k* values of compounds were determined using C-8, C-18 and IAM stationary phases. The mobile phases consisted of buffer mixed with various amounts of methanol or acetonitrile to give pH 7.4. Linear relationships were obtained between the log *k* and *φ* in the mobile phase in the whole studied ranges for all stationary phases. Generally, for semicarbazide derivatives and IAM chromatography, a larger content of water in the mobile phase can be used (Tables 1, 2). For some compounds log *k*_w_ values on IAM were determined experimentally. The chromatograms of compound **12a** are shown in Fig. 2.

**Table 1:** Chromatographic parameters: log *k*_w_ (intercept) and −*S* (slope) of the linear dependences in Eq. 1 obtained by RP-8 and RP-18 chromatography

	RP-8 HPLC					RP-18 HPLC				
	−*S*	Intercept	*r* ^2^	*φ* MeOH	*n*	−*S*	Intercept	*r* ^2^	*φ* MeOH	*n*
**1a**	3.978	1.946	0.970	0.70–0.25	7	1.880	0.783	0.982	0.75–0.35	7
**2a**	3.847	2.144	0.979	0.70–0.35	5	2.279	1.279	0.981	0.75–0.35	7
**3a**	3.449	1.022	0.984	0.70–0.20	6	1.155	0.089	0.984	0.75–0.35	7
**4a**	3.728	1.717	0.981	0.70–0.25	7	2.065	0.871	0.982	0.75–0.30	8
**5a**	2.967	0.797	0.991	0.55–0.10	9	0.910	−0.074	0.901	0.75–0.30	7
**6a**	3.371	1.563	0.992	0.70–0.20	7	1.959	0.864	0.992	0.75–0.30	7
**7a**	4.872	2.443	0.984	0.55–0.30	6	2.365	1.280	0.960	0.75–0.35	6
**8a**	2.919	0.799	0.991	0.70–0.10	9	0.987	0.014	0.984	0.75–0.30	7
**9a**	2.948	0.721	0.990	0.70–0.10	9	1.257	0.155	0.972	0.75–0.30	7
**10a**	2.293	0.581	0.990	0.70–0.10	9	0.854	−0.154	0.907	0.75–0.30	7
**11a**	3.725	1.783	0.977	0.70–0.30	6	1.452	0.479	0.934	0.75–0.30	7
**12a**	3.422	1.682	0.985	0.70–0.25	7	1.506	0.471	0.857	0.75–0.30	7
**13a**	3.386	1.234	0.992	0.70–0.10	9	1.451	0.446	0.988	0.75–0.35	6
**14a**	4.398	1.705	0.970	0.70–0.20	8	0.590	−0.352	0.949	0.75–0.30	7
**15a**	4.401	1.998	0.930	0.70–0.25	7	1.406	0.270	0.953	0.75–0.30	6
**1b**	4.140	2.350	0.972	0.70–0.35	5	2.392	1.341	0.963	0.75–0.40	6
**2b**	4.153	2.434	0.978	0.70–0.35	5	2.623	1.545	0.965	0.75–0.40	6
**3b**	3.301	1.324	0.973	0.70–0.20	7	1.353	0.335	0.973	0.75–0.35	7
**4b**	3.841	2.139	0.974	0.70–0.25	5	2.507	1.439	0.964	0.75–0.40	6
**5b**	3.539	1.550	0.981	0.55–0.20	7	1.591	0.540	0.931	0.75–0.30	7
**6b**	3.601	1.987	0.975	0.80–0.30	7	2.670	1.514	0.975	0.75–0.40	6
**7b**	5.124	2.893	0.959	0.70–0.40	6	3.268	2.069	0.965	0.75–0.40	6
**8b**	3.637	1.661	0.980	0.70–0.20	8	1.758	0.745	0.892	0.75–0.35	7
**9b**	3.417	1.551	0.979	0.70–0.20	8	1.757	0.758	0.995	0.75–0.30	7
**10b**	2.711	0.874	0.975	0.70–0.10	9	1.767	0.36	0.853	0.75–0.30	7
**11b**	4.591	2.172	0.981	0.70–0.30	6	1.905	0.862	0.985	0.75–0.30	7
**12b**	4.829	2.244	0.981	0.70–0.30	6	2.331	0.977	0.899	0.75–0.30	7
**13b**	3.793	1.923	0.973	0.70–0.30	6	2.211	1.126	0.925	0.75–0.30	6
**14b**	6.725	3.038	0.979	0.70–0.30	6	1.974	0.792	0.991	0.75–0.30	7
**15b**	6.305	3.311	0.954	0.70–0.35	5	2.821	1.531	0.902	0.75–0.30	7

**Table 2:** Chromatographic parameters: log *k*_w_ (intercept) and −*S* (slope) of the linear dependences in Eq. 1 obtained by IAM chromatography and log *P* values

	IAM HPLC					log *P*
	−*S*	Intercept	*r* ^2^	*φ* ACN	*n*	
**1a**	5.094	1.257	0.995	0.30–0.05	6	0.91
**2a**	5.358	1.847	0.993	0.30–0.10	5	1.53
**3a**	3.514	0.067	0.901	0.40–0.15	6	−0.63
**4a**	4.077	0.825	0.997	0.30–0.00	7	0.77
**5a**	2.458	0.097	0.955	0.25–0.00	6	−0.27
**6a**	4.626	1.230	0.995	0.50–0.15	8	1.90
**7a**	5.061	1.522	0.995	0.30–0.05	6	2.39
**8a**	2.453	0.003	0.868	0.30–0.00	7	0.58
**9a**	2.783	0.101	0.993	0.30–0.00	7	0.09
**10a**	2.581	0.206	0.965	0.30–0.00	7	−0.39
**11a**	5.227	1.137	0.997	0.30–0.05	6	0.47
**12a**	4.588	0.740	0.993	0.30–0.00	7	0.79
**13a**	3.318	0.318	0.986	0.30–0.00	6	0.95
**14a**	6.727	1.459	0.998	0.30–0.05	6	0.21
**15a**	7.091	1.725	0.996	0.30–0.05	6	1.52
**1b**	5.878	1.543	0.996	0.30–0.05	6	2.04
**2b**	5.519	1.705	0.996	0.30–0.10	5	2.65
**3b**	4.228	0.441	0.985	0.40–0.15	6	0.50
**4b**	5.211	1.391	0.998	0.30–0.05	6	1.90
**5b**	4.062	0.513	0.995	0.30–0.00	7	0.85
**6b**	4.761	1.243	0.998	0.50–0.15	8	3.03
**7b**	5.900	1.760	0.997	0.30–0.10	5	3.52
**8b**	4.274	0.712	0.995	0.30–0.00	7	1.70
**9b**	3.782	0.584	0.908	0.30–0.00	7	1.22
**10b**	3.994	0.158	0.998	0.30–0.00	7	0.74
**11b**	5.374	0.985	0.986	0.30–0.05	6	1.59
**12b**	5.924	1.056	0.994	0.30–0.00	7	1.92
**13b**	4.593	0.935	0.996	0.30–0.00	7	2.08
**14b**	6.902	1.322	0.994	0.30–0.05	6	2.47
**15b**	7.684	1.913	0.997	0.30–0.10	5	3.78

The equations of the straight lines and statistics for three techniques are reported in Tables 1 and 2. As follows from the tables the absolute values of the slopes |*S*| are the highest for the IAM stationary phase for both semicarbazides and 3*H*-1,2,4-triazol-3-ones. In the case of RP-8 and RP-18 chromatography, the log *k*_w_ values for the linear structures (**1a**–**15a**) are lower than for the cyclic ones (**1b**–**15b**).

Biagi et al. [25] found that for closely congeneric compounds, the ratio of the intercept (log *k*_w_) to the slope (*S*) in Eq. 1 is constant. The relationships between these parameters of semicarbazides (**a**) for RP-8, 18 and IAM chromatography are expressed by the following equations:

2 $$ {\log}\;k_{\rm{w(C8 {(\mathbf{a})})}}= 0.774\;( \pm 0.106)\;(-S)_{\rm{C8 {(\mathbf{a}})}}-1.295\;( \pm 0.383) \quad n=15,r=0.896,s=0. 266,F=52.9,df(1.13) $$

3 $$ {\log}\;k_{\rm{w(C18 {(\mathbf{a})})}}=0.928\;( \pm 0.031)\;(-S)_{\rm{C18 {(\mathbf{a}})}}-0.940\;( \pm 0.049) \quad n=15,r = 0.992,s=0.066,F=918.3,df(1.13) $$

4 $$ {\log}\;k_{\rm{w(IAM {(\mathbf{a})})}}=0.407\;( \pm 0.051)\;(-S)_{\rm{IAM {(\mathbf{a}})}}-0.927\;( \pm 0.233) \quad n=15,r = 0.911,s=0.284,F=63.3,df(1.13). $$

In the case of their cyclic analogues 3*H*-1,2,4-triazol-3-ones (**b**), these relations are described by Eqs. 5–7:

5 $$ {\log}\;k_{\rm{w(C8 {(\mathbf{b})})}}= 0.547\;( \pm 0.065)\;(-S)_{\rm{C8 {(\mathbf{b}})}}-0.288\;( \pm 0.286) \quad n=15,r=0.918,s=0.272,F=69.9,df(1.13) $$

6 $$ {\log}\;k_{\rm{w(C18 {(\mathbf{b})})}}= 0.921\;( \pm 0.064)\;(-S)_{\rm{C18 {(\mathbf{b}})}}-0.963\;( \pm 0.145) \quad n=15,r=0.969,s=0.127,F=205.3,df(1.13) $$

7 $$ {\log}\;k_{\rm{w(IAM {(\mathbf{b})})}}= 0.387\;( \pm 0.074)\;(-S)_{\rm{IAM {(\mathbf{b}})}}-0.928\;( \pm 0.395) \quad n=15,r=0.821,s=0.313,F=27.1,df(1.13). $$

**Fig. 3:**
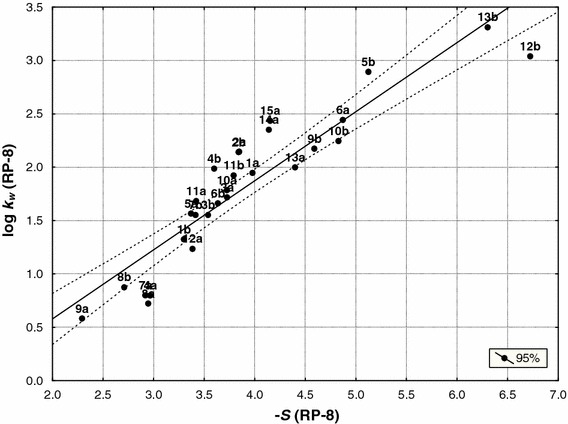
Relationship between the intercept (log *k*_w_) and slopes (−*S*) of both groups of compounds (**a** and **b**) obtained by RP-8 stationary phase (Eq. 8)

**Fig. 4:**
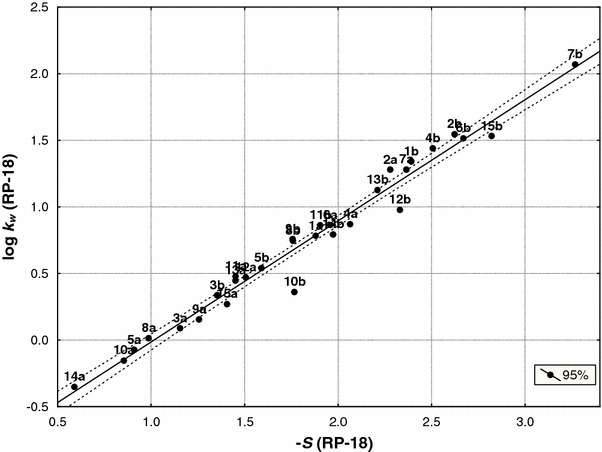
Relationship between the intercept (log *k*_w_) and slopes (−*S*) of both groups of compounds (**a** and **b**) obtained by RP-18 stationary phase (Eq. 9)

**Fig. 5:**
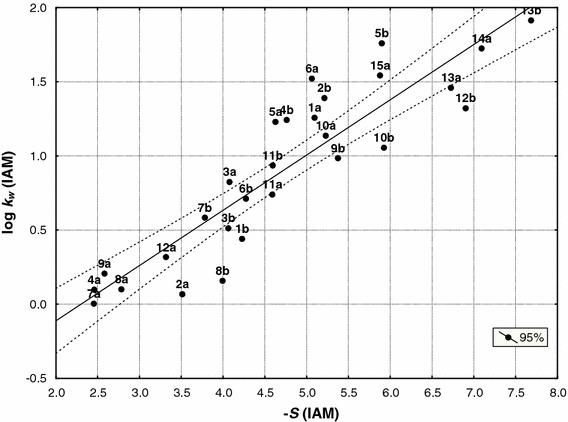
Relationship between the intercept (log *k*_w_) and slopes (−*S*) of both groups of compounds (**a** and **b**) obtained by IAM stationary phase (Eq. 10)

The best results were obtained for the C-18 stationary phase (Eqs. 3, 6). However, in all cases congenerity of compounds was confirmed. The slopes of Eqs. 2 and 5, 3 and 6, as well as 4 and 7 are similar. This may indicate the same retention mechanism of both studied compounds in these chromatographic systems. The relationships between the intercept (log *k*_w_) and the slope (–*S*) of both groups of compounds for three chromatographic systems are presented in Figs. 3, 4 and 5.

They are expressed by the following equations:

8 $$ {\log}\;k_{\rm{w(C8 {(\mathbf{a,b})})}}= 0.648\;( \pm 0.057)\;(-S)_{\rm{C8 {(\mathbf{a,b}})}}-0.750\;( \pm 0.048) \quad n=30,r=0.907,\,s=0.294,\,F=130.2,\,df(1.28) $$

9 $$ {\log}\;k_{\rm{w(C18 {(\mathbf{a,b})})}}= 0.901\;( \pm 0.028)\;(-S)_{\rm{C18 {(\mathbf{a,b}})}}-0.924\;( \pm 0.048) \quad n=30,\,r=0.987,\,s=0.097,\,F=1,040.2,\,df(1.28) $$

10 $$ {\log}\;k_{\rm{w(IAM {(\mathbf{a,b})})}}= 0.373\;( \pm 0.035)\;(-S)_{\rm{IAM {(\mathbf{a,b}})}}-0.857\;( \pm 0.172) \quad n=28,\,r=0.902,\,s=0.256,\,F=113.2,\,df(1.26). $$

Compounds **2a** and **2b** are outliers in IAM chromatography. The equations show that semicarbazides and corresponding 3*H*-1,2,4-triazol-3-ones are a set of congeneric compounds [25, 26]. Therefore, they were analyzed together during further studies.

The relationships between the log *k*_w_ values obtained by different chromatographic techniques are not very powerful. It can be assumed that compounds **14** and **15** (with two semicarbazide moieties or two triazole rings) are outliers. Under this assumption, the relationships between the intercepts (log *k*_w_) are described in the following way:

11 $$ {\log}\;k_{\rm{w(C18 {(\mathbf{a,b})})}}= 0.853\;( \pm 0.071)\;{\log}\;k_{\rm{w(C8 {(\mathbf{a,b})})}}-0.654\;( \pm 0.126) \quad n=26,r=0.926,s=0.218,F=144.5,df(1.24) $$

12 $$ {\log}\;k_{\rm{w(C18 {(\mathbf{a,b})})}}= 0.692\;( \pm 0.102)\;{\log}\;k_{\rm{w(IAM {(\mathbf{a,b})})}}+0.563\;( \pm 0.094) \quad n=26,r=0.902,s=0.249,F=105.1,df(1.24) $$

13 $$ {\log}\;k_{\rm{w(C8 {(\mathbf{a,b})})}}= 0.979\;( \pm 0.083)\;{\log}\;k_{\rm{w(IAM {(\mathbf{a,b})})}}+0.832\;( \pm 0.085) \quad n=26,r=0.924,s=0.240,F=139.9,df(1.24). $$

The weakest correlation was found between the most hydrophobic phase and the most polar one (Eq. 12).

For comparison, the theoretical values of log *P* were calculated according to the fragmentation method introduced by Crippen [27]. The analysis of both groups of compounds gives the following equation:

14 $$ {\log}\;k_{\rm{w(C18)}}=0.451\;( \pm 0.058)\;{\log}\;P+0.105\;(\pm 0.106)\quad n=26,r=0.937,s=0.201,F=174.1,df(1.24) $$

15 $$ {\log}\;k_{\rm{w(C8)}}=0.533\;( \pm 0.059)\;{\log}\;P+1.061\;(\pm 0.106)\quad n=30,r=0.862,s=0.355,F=81.0,df(1.28). $$

Compounds **14** and **15** are outliers in Eq. 14. In the case of IAM chromatography very poor correlation was obtained (*r* = 0.753, *n* = 30).

Markedly different chromatographic behaviour of solutes on the IAM chromatography is a result of some differences in the stationary phase structure [28]. Generally, the correlation between the logarithms of the retention factor determined on the IAM columns log *k*_w(IAM)_ and the reference parameter of hydrophobicity, log *D* as well as log *k*_w(C18)_ (log*P*) is not large [29]. However, the log *k*_w(IAM)_ values appeared to be a better predictor of bioactivity than log *D* for several classes of drugs [29, 30].

As the transformation of semicarbazides into 3*H*-1,2,4-triazol-3-ones is associated with the elimination of water, the differences between log *k*_w(b)_ and log *k*_w(a)_ values, Δ log *k*_w_, were calculated. Table 3 shows that the lipophilicity differences between the corresponding derivatives in RP-18 chromatography, Δ log *k*_w(C18)_, are in the range about 0.5–0.7. Higher values (about 1.2) for compounds **14** and **15** were found. They are formed by the elimination of two water molecules from the linear substrates (Scheme 1), so the lipophilicity differences are about twice as large. A similar trend for RP-8 chromatography was observed (Table 3). This finding reflects the synthesis mechanism of compounds of structure **b** from **a**. At the same time it shows that the elimination of water from a molecule reduces its lipophilicity as well as absolute values of the specific hydrophobic surface, and the ratio of the intercept (log *k*_w_) to the slope (−*S*) of compounds is constant in both groups. The obtained results are in accordance with the fragmental method used for log *P* calculations [27]. The differences between log *P* values of compound structures **b** and **a** are equal 1.13 (1.12) for analogues **1**–**13** and 2.26 for **14** and **15**. The differences between the log *k*_w(IAM(**b**))_ and log *k*_w(IAM(**a**))_ values, Δ log *k*_w(IAM)_, are varied (Table 3).

**Table 3:** The lipophilicity differences between the 3*H*-1,2,4-triazol-3-ones and the corresponding semicarbazides (Δ log *k*_w_) obtained by different chromatographic systems

	Δ log *k*_w(8)_^a^	Δ log *k*_w(C18)_^b^	Δ log *k*_w(IAM)_^c^
**1a**,**b**	0.404	0.558	0.286
**2a**,**b**	0.290	0.266	−0.142
**3a**,**b**	0.301	0.246	0.373
**4a**,**b**	0.422	0.568	0.566
**5a**,**b**	0.754	0.614	0.415
**6a**,**b**	0.423	0.650	0.013
**7a**,**b**	0.450	0.789	0.238
**8a**,**b**	0.862	0.731	0.709
**9a**,**b**	0.830	0.603	0.483
**10a**,**b**	0.293	0.514	−0.048
**11a**,**b**	0.389	0.383	−0.152
**12a**,**b**	0.563	0.506	0.316
**13a**,**b**	0.689	0.680	0.617
**14a**,**b**	1.333	1.144	−0.138
**15a**,**b**	1.313	1.261	0.189

^a^Δ log *k*_w(C8)_ = log *k*_w(C8(**b**))_ − log *k*_w(C8(**a**))_

^b^Δ log *k*_w(C18)_ = log *k*_w(C18(**b**))_ − log *k*_w(C18(**a**))_

^c^Δ log *k*_w(IAM)_ = log *k*_w(IAM(**b**))_ − log *k*_w(IAM(**a**))_

**Fig. 6:**
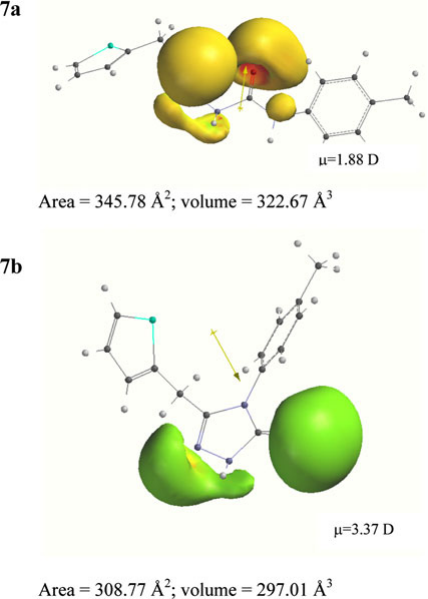
Optimised structures of **7a** and **7b** and the MEPs of the electrostatic potential profile at −84 kJ mol^−1^

To compare the structures of compounds quantum mechanical calculations were carried out with the semi-empirical model at the PM3 basis set. In Fig. 6 compounds **7a** and **7b** are shown as representative analogues of the analysed groups.

As follows from Fig. 6 elimination of water from an **a** molecule remarkably changes its shape, flexibility and dipole moment, and decreases both the area and volume. Of course, changes of individual compounds are also a function of the substituents. The MEPs, the electrostatic potential profile of compounds, show that the most negative potential of compound structure **a** is located on four heteroatoms of the semicarbazide moiety (without taking the substituent into account). In the cyclic analogues **b** it covers mainly two oxygen atoms. The area and volume reduction of **b** compared to **a** is associated with reduction of the polar local area of molecules. It causes stronger interactions with the hydrophobic octadecyl or octyl stationary phases and increases lipophilicity.

In conclusion, new semicarbazides and corresponding 1,2,4-triazol-3-one derivatives have been synthesized. In RP-8, RP-18 and IAM chromatography, the dependences of log *k* versus the concentration of an organic modifier (*φ*) in the mobile phase were linear. The constant ratio of the intercept (log *k*_w_) to the slope (−*S*) of compounds showed that the compounds of both groups are congeneric according to Biagi. The differences in the retention behaviour of compounds of structures **b** and **a** in C-18 and C-8 chromatography reflect the synthesis method of 1,2,4-triazol-3-ones from linear substrates connected with the elimination of water from a molecule. The good correlation coefficient between the log *k*_w_ values obtained by octadecyl and octyl phases suggests that these parameters can be used independently in the evaluation of compounds under consideration.

## Experimental

Melting points were determined in a Fisher-Johns block. Elemental analyses (C, H, N) were conducted using an Elemental Analyser CHN; their results were found to be in good agreement with the calculated values. IR spectra were recorded from KBr discs using a Specord IR-75 spectrophotometer. ^1^H NMR spectra were recorded on a Bruker AC 300F instrument (300 MHz) in DMSO-*d*_*6*_ with TMS as an internal standard. ^13^C NMR spectra were recorded on a Bruker AC 250F instrument (62.5 MHz) in DMSO-*d*_*6*_ with TMS as an internal standard. Chemicals were purchased from Alfa-Aesar or Merck. The purity of the obtained compounds was checked by TLC on aluminum oxide 60 F_254_ plates (Merck) in a CHCl_3_/C_2_H_5_OH (10:1 and 10:2) solvent system with UV or iodine visualization. Compounds **1**–**4**, **6**–**8**, **11a**–**13a** and **14** were synthesized and characterized earlier [31–35].

### Synthesis of 1,4-disubstituted semicarbazides

To 10 mmol of appropriate carboxylic acid hydrazide dissolved in 10 cm^3^ of diethyl ether 10 mmol isocyanate was added. The mixture was kept at room temperature for 24 h. The precipitate was filtered and crystallized from ethanol.

#### *1*-*Methyl*-*1H*-*pyrrole*-*2*-*acetic acid 2*-*[(2*-*propenylamino)carbonyl]hydrazide* (**5a**, C_11_H_16_N_4_O_2_)

Yield 1.96 g (83%); m.p.: 137–139 °C; IR (KBr): $\overline{\nu }$ = 3,279, 3,192, 3,034, 1,701, 1,649, 1,444 cm^−1^; ^1^H NMR (300 MHz, DMSO-*d*_*6*_): *δ* = 3.32 (s, 2H), 3.52 (s, 3H), 3.61–3.65 (m, 2H), 4.98–5.15 (m, 2H), 5.72–5.84 (m, 1H), 6.85–7.63 (m, 3H, pyrrole-H), 7.63 (s, NH), 7.78 (s, NH), 9.60 (s, NH) ppm; ^13^C NMR (62.5 MHz, DMSO-*d*_*6*_): *δ* = 31.45 (CH_2_), 33.52 (CH_3_), 41.42 (CH_2_), 106.01 (CH), 107.84 (C_arom_), 114.40 (CH_2_), 121.82, 126.09, 136.28 (3 × C_arom_), 157.91 (C=O), 169.26 (C=O) ppm.

#### *Pyridine*-*2*-*carboxylic acid 2*-*[(2*-*propenylamino)carbonyl]hydrazide* (**9a**, C_10_H_12_N_4_O_2_)

Yield 1.76 g (80%); m.p.: 195–197 °C; IR (KBr): $\overline{\nu }$ = 3,261, 3,013, 2,814, 1,697, 1,651, 1,487 cm^−1^; ^1^H NMR (300 MHz, DMSO-*d*_*6*_): *δ* = 3.63–3.72 (m, 2H), 4.98–5.18 (m, 2H), 5.71–5.86 (m, 1H), 6.59 (s, NH), 7.56–8.08 (m, 4H, pyridine-H), 8.72 (s, NH), 10.26 (s, NH) ppm; ^13^C NMR (62.5 MHz, DMSO-*d*_*6*_): *δ* = 13.31 (CH_2_), 61.74 (CH_2_), 113.06 (CH), 123.12, 130.03, 143.23, 148.01, 152.48 (5 × C_arom_), 154.21 (C=O), 167.73 (C=O) ppm.

#### *Pyridine*-*4*-*acetic acid 2*-*[(2*-*propenylamino)carbonyl]hydrazide* (**10a**, C_11_H_14_N_4_O_2_)

Yield 1.62 g (69%); m.p.: 130–131 °C; IR (KBr): $\overline{\nu }$ = 3,309, 3,267, 2,960, 1,644, 1,598, 1,455 cm^−1^; ^1^H NMR (300 MHz, DMSO-*d*_*6*_): *δ* = 3.49 (s, 2H), 3.56–3.70 (m, 2H), 5.00–5.15 (m, 2H), 5.72–5.85 (m, 1H), 6.53 (t, *J* = 5.6 Hz, NH), 7.20–8.49 (m, 4H, pyridine-H), 7.85 (s, NH), 9.82 (s, NH) ppm; ^13^C NMR (62.5 MHz, DMSO-*d*_*6*_): *δ* = 40.00 (CH_2_), 40.11 (CH_2_), 113.12 (CH_2_), 123.26 (CH), 123.82, 143.26, 147.78, 147.96 (5 × C_arom_), 156.62 (C=O), 167.68 (C=O) ppm.

#### *Propanoic acid 1,1′*-*[2,2′*-*[methylenebis(4,1*-*phenyleneiminocarbonyl)]dihydrazide]* (**15a**, C_21_H_26_N_6_O_4_)

Yield 3.49 g (82%); m.p.: 356–358 °C; IR (KBr): $\overline{\nu }$ = 3,304, 3,032, 2,977, 1,650, 1,463 cm^−1^; ^1^H NMR (300 MHz, DMSO-*d*_*6*_): *δ* = 1.95–2.07 (m, 6H), 2.10–2.35 (m, 4H), 3.80 (s, 2H), 7.06–7.39 (m, 8H, phenyl-H), 7.90 (s, 2NH), 8.64 (s, 2NH), 9.54 (s, 2NH) ppm; ^13^C NMR (62.5 MHz, DMSO-*d*_*6*_): *δ* = 7.01 (2 × CH_3_), 8.26 (2 × CH_2_), 25.06 (CH_2_), 117.38, 118.72, 127.42, 133.80, 136.07 (12 × C_arom_), 154.20 (2 × C=O), 170.80 (2 × C=O) ppm.

### Synthesis of 4,5-disubstituted 2,4-dihydro-3H-1,2,4-triazol-3-one derivatives

A mixture of 10 mmol of semicarbazide derivatives **a** and 50 cm^3^ of 2% aqueous solution of sodium hydroxide was refluxed for 20 h. After cooling, the solution was neutralized with dilute hydrochloric acid. The precipitate was filtered off and then crystallized from ethanol.

#### *2,4*-*Dihydro*-*5*-*[(1*-*methyl*-*1H*-*pyrrol*-*2*-*yl)methyl]*-*4*-*(2*-*propenyl)*-*3H*-*1,2,4*-*triazol*-*3*-*one* (**5b**, C_11_H_14_N_4_O)

Yield 1.65 g (76%); m.p.: 110–112 °C; IR (KBr): $\overline{\nu }$ = 3,283, 3,088, 3,015, 2,854, 1,697, 1,647, 1,461 cm^−1^; ^1^H NMR (300 MHz, DMSO-*d*_*6*_): *δ* = 3.49 (s, 3H), 3.83 (s, 2H), 4.12–4.14 (m, 2H), 4.92–5.13 (m, 2H), 5.66–5.83 (m, 1H), 5.88–6.66 (m, 3H, pyrrol-H), 11.52 (s, NH) ppm; ^13^C NMR (62.5 MHz, DMSO-*d*_*6*_): *δ* = 22.17 (CH_2_), 32.04 (CH_3_), 40.86 (CH_2_), 104.95 (CH), 106.51 (C_arom_), 115.14 (CH_2_), 121.14, 123.62, 131.36, 143.80 (5 × C_arom_), 153.38 (C=O) ppm.

#### *2,4*-*Dihydro*-*4*-*(2*-*propenyl)*-*5*-*(pyridin*-*2*-*yl)*-*3H*-*1,2,4*-*triazol*-*3*-*one* (**9b**, C_10_H_10_N_4_O)

Yield 1.37 g (68%); m.p.: 183–184 °C; IR (KBr): $\overline{\nu }$ = 3,180, 3,014, 2,953, 2,814, 1,964, 1,640, 1,454 cm^−1^; ^1^H NMR (300 MHz, DMSO-*d*_*6*_): *δ* = 3.50–3.59 (m, 2H), 4.11–4.77 (m, 2H), 5.11–5.16 (m, 1H), 7.02–7.30 (m, 4H, pyridine-H), 11.05 (s, NH) ppm; ^13^C NMR (62.5 MHz, DMSO-*d*_*6*_): *δ* = 39.00 (CH_2_), 42.29 (CH_2_), 114.71 (CH), 132.20, 136.00, 142.62, 145.61, 147.47 (6 × C_arom_), 153.75 (C=O) ppm.

#### *2,4*-*Dihydro*-*4*-*(2*-*propenyl)*-*5*-*[(pyridin*-*4*-*yl)methyl]*-*3H*-*1,2,4*-*triazol*-*3*-*one* (**10b**, C_11_H_12_N_4_O)

Yield 1.60 g (74%); m.p.: 255–257 °C; IR (KBr): $\overline{\nu }$ = 3,180, 3,065, 2,790, 2,654, 1,703, 1,639, 1,457 cm^−1^; ^1^H NMR (300 MHz, DMSO-*d*_*6*_): *δ* = 4.25 (s, 2H), 4.21–4.27 (m, 2H), 4.93–5.09 (m, 2H), 5.71–5.84 (m, 1H), 7.95–8.88 (m, 4H, pyridine-H), 11.79 (s, NH) ppm; ^13^C NMR (62.5 MHz, DMSO-*d*_*6*_): *δ* = 29.80 (CH_2_), 39.05 (CH_2_), 40.81 (CH_2_), 115.45 (CH), 126.38, 131.16, 139.92, 142.93 (6 × C_arom_), 154.29 (C=O) ppm.

#### *4*-*Cyclohexyl*-*2,4*-*dihydro*-*5*-*[(pyridin*-*4*-*yl)methyl]*-*3H*-*1,2,4*-*triazol*-*3*-*one* (**11b**, C_14_H_18_N_4_O)

Yield 2.06 g (80%) m.p.: 237–238 °C; IR (KBr): $\overline{\nu }$ = 3,231, 3,075, 2,916, 1,687, 1,564 cm^−1^; ^1^H NMR (300 MHz, DMSO-*d*_*6*_): *δ* = 0.96–2.06 (m, 10H), 3.48–3.61 (m, 1H), 4.00 (s, 2H), 7.27–8.53 (m, 4H, pyridine-H), 11.48 (s, NH) ppm; ^13^C NMR (62.5 MHz, DMSO-*d*_*6*_): *δ* = 24.53 (CH_2_), 25.14 (2 × CH_2_), 28.97 (2 × CH_2_), 30.94 (CH_2_), 53.97 (CH), 123.92, 144.96, 145.06, 149.74 (6 × C_arom_), 154.69 (C=O) ppm.

#### *4*-*(4*-*Ethoxyphenyl)*-*2,4*-*dihydro*-*5*-*[(pyridin*-*4*-*yl)methyl]*-*3H*-*1,2,4*-*triazol*-*3*-*one* (**12b**, C_16_H_16_N_4_O_2_)

Yield 2.10 g (71%); m.p.: 180–181 °C; IR (KBr): $\overline{\nu }$ = 3,231, 3,074, 2,917, 1,678, 1,475 cm^−1^; ^1^H NMR (300 MHz, DMSO-*d*_*6*_): *δ* = 1.32 (t, *J* = 6.9 Hz, 3H), 3.82 (s, 2H), 4.05 (q, *J* = 6.9 Hz, 2H), 6.94–7.07 (m, 4H, phenyl-H), 7.14–8.41 (m, 4H, pyridine-H), 11.76 (s, NH) ppm; ^13^C NMR (62.5 MHz, DMSO-*d*_*6*_): δ = 14.49 (CH_3_), 31.23 (CH_2_), 63.36 (CH_2_), 114.86, 124.06, 124.96, 128.82, 144.42, 144.99, 149.22, 154.54 (12 × C_arom_), 158.42 (C=O) ppm.

#### *2,4*-*Dihydro*-*5*-*(phenylmethyl)*-*4*-*(2*-*propenyl)*-*3H*-*1,2,4*-*triazol*-*3*-*one* (**13b**, C_12_H_13_N_3_O)

Yield 1.57 g (73%); m.p.: 105–108 °C; IR (KBr): $\overline{\nu }$ = 3,143, 3,064, 2,780, 1,702, 1,641, 1,449 cm^−1^; ^1^H NMR (300 MHz, DMSO-*d*_*6*_): *δ* = 3.30–3.45 (m, 2H), 4.10 (s, 2H), 4.66–5.05 (m, 2H), 5.40–5.70 (m, 1H), 6.93–7.32 (m, 5H, phenyl-H), 11.64 (s, NH) ppm; ^13^C NMR (62.5 MHz, DMSO-*d*_*6*_): δ = 31.44 (CH_2_), 40.05 (CH_2_), 116.55 (CH_2_), 126.85 (CH), 128.54, 128.64, 132.48, 135.19, 146.23 (7 × C_arom_), 154.81 (C=O) ppm.

#### *4,4′*-*(Methylenedi*-*4,1*-*phenylene)bis(5*-*ethyl*-*2,4*-*dihydro*-*3H*-*1,2,4*-*triazol*-*3*-*one)* (**15b**, C_21_H_22_N_6_O_2_)

Yield 2.61 g (68%); m.p.: 308–310 °C; IR (KBr): $\overline{\nu }$ = 3,176, 3,066, 2,955, 1,698, 1,580, 1,417 cm^−1^; ^1^H NMR (300 MHz, DMSO-*d*_*6*_): *δ* = 1.00 (t, *J* = 4.6 Hz, 6H), 2.36 (q, *J* = 7.4 Hz, 4H), 3.32 (s, 2H), 7.05–7.94 (m, 8H, phenyl-H), 11.59 (s, 2NH) ppm; ^13^C NMR (62.5 MHz, DMSO-*d*_*6*_): *δ* = 8.30 (2 × CH_3_), 17.92 (2 × CH_2_), 39.05 (CH_2_), 126.05, 128.32, 139.98 (14 × C_arom_), 146.54 (2 × C=O) ppm.

### UV-Vis spectroscopy

UV-Vis spectra were recorded in water (buffer)-methanol (1:1) solution on a UV-160A Shimadzu Spectrophotometer. Quartz cuvettes (1 cm) were used for measurements.

### Liquid chromatography

HPLC was carried out using a liquid chromatograph (Knauer, Berlin, Germany) with a dual pump, a 20-mm^3^ simple injection valve and a UV–visible detector at 254 nm. The samples were prepared as solutions in methanol.

### RP-8 chromatography

A Hypersil MOS2 C8 (5 μm, 150 × 4.6 mm) column was used as the stationary phase. The mobile phase consisted of different volume mixtures of methanol and 20 mM acetate buffer (KCl) as the aqueous phase to give pH 7.4. The methanol concentration ranged from 0.10 to 0.70 at 0.1 (0.05) intervals. The flow rate was 1 cm^3^ min^−1^ at room temperature. The retention time of an unretained solute (*t*_0_) was determined by the injection of a small amount of acetone dissolved in water.

### RP-18 chromatography

A Hypersil Gold C18 (3 μm, 100 × 3 mm) column was used as the stationary phase. The mobile phase consisted of different volume mixtures of methanol and 20 mM phosphate buffer as the aqueous phase to give pH 7.4. The methanol concentration ranged from 0.30 to 0.75, depending on the structure of compound, at 0.05 (0.1) intervals. The flow rate was 0.5 cm^3^ min^−1^ at room temperature. The retention time of an unretained solute (*t*_0_) was determined by the injection of a small amount of acetone dissolved in water.

### IAM chromatography

A Rexchrom IAM.PC.DD2 (12 μm, 100 × 4.6 mm, 300 Å) (Regis Technologies) column was used as the stationary phase. The mobile phase consisted of different volume mixtures of acetonitrile and 20 mM phosphate buffer as the aqueous phase to give pH 7.4 (0.02 M KH_2_PO_4_, Na_2_HPO_4_, and 0.15 M KCl). The acetonitrile concentration ranged from 0 to 0.50, depending on the structure of compound, at 0.05 intervals. The flow rate was 1 cm^3^ min^−1^ at room temperature. The retention time of an unretained solute (*t*_0_) was determined by the injection of a small amount of citric acid dissolved in water [36, 37].

### Computational methods

The 3D structure of each compound was built up from the fragment library in the PC SPARTAN Pro ver 1.08 molecular modeling program, and it was fully geometry-optimized at the semi-empirical PM3 level [38]. The energy-minimized structure was subjected to conformational analysis implemented in SPARTAN. Conformational analysis was carried out through systemic conformation option. The conformers of the lowest energy were compared. The log *P* values were calculated using ChemDraw Ultra 10.0 [39].

### Statistic analysis

The coefficients in the regression equations were calculated by the multiple regression analysis program Statistica, version 7.1 [40]. Statistical significance of the regression equation was tested by the correlation coefficient (*r*), the standard error of estimate (*s*) and the variance ratio (*F*) at specified degrees of freedom (*df*), *n* number of compounds.
